# Nondestructive Identification of Eggshell Cracks Using Hyperspectral Imaging Combined with Attention-Enhanced 3D-CNN

**DOI:** 10.3390/foods14244183

**Published:** 2025-12-05

**Authors:** Hao Li, Aoyun Zheng, Chaoxian Liu, Jun Huang, Yong Ma, Huanjun Hu, You Du

**Affiliations:** 1 School of Mathematics and Computer Science, Wuhan Polytechnic University, Wuhan 430048, China; lihao@whpu.edu.cn (H.L.); cx_leo@whpu.edu.cn (C.L.); huhuanjun@whpu.edu.cn (H.H.); 2 Electronic Information School, Wuhan University, Wuhan 430072, China; junhwong@whu.edu.cn (J.H.); mayong@whu.edu.cn (Y.M.); dy0815@whu.edu.cn (Y.D.)

**Keywords:** hyperspectral imaging, 3D convolutional neural network, attention mechanism, eggshell crack, residual modules

## Abstract

Eggshell cracks are a critical factor affecting egg quality and food safety, with traditional detection methods often struggling to detect fine cracks, especially under multi-colored shells and complex backgrounds. To address this issue, we propose a non-destructive detection approach based on an enhanced three-dimensional convolutional neural network (3D-CNN), named 3D-CrackNet, integrated with hyperspectral imaging (HSI) for high-precision identification and localization of eggshell cracks. Operating within the 1000–2500 nm spectral range, the proposed framework employs spectral preprocessing and optimal band selection to improve discriminative feature representation. A residual learning module is incorporated to mitigate gradient degradation during deep joint spectral-spatial feature extraction, while a parameter-free SimAM attention mechanism adaptively enhances crack-related regions and suppresses background interference. This architecture enables the network to effectively capture both fine-grained spatial textures and contiguous spectral patterns associated with cracks. Experiments on a self-constructed dataset of 400 egg samples show that 3D-CrackNet achieves an F1-score of 75.49% and an Intersection over Union (IoU) of 60.62%, significantly outperforming conventional 1D-CNN and 2D-CNN models. These findings validate that 3D-CrackNet offers a robust, non-destructive, and efficient solution for accurately detecting and localizing subtle eggshell cracks, demonstrating strong potential for intelligent online egg quality grading and micro-defect monitoring in industrial applications.

## 1. Introduction

Eggshell cracks significantly compromise both the visual quality and structural integrity of eggs, leading to moisture loss, nutrient degradation, and increased risk of microbial contamination (e.g., *Salmonella*). Such defects not only shorten shelf life and reduce commercial value, but may also cause food safety incidents [1,2]. Current eggshell crack detection methods mainly include manual inspection, acoustic techniques, and machine vision. However, manual inspection is prone to subjective bias and fatigue, resulting in low efficiency and low speed [3]; acoustic detection is susceptible to interference from industrial vibrations and noise, and it may cause secondary damage [4]; although machine vision offers high speed, its ability to detect invisible or micro-scale cracks, especially under varying shell colors, dirt contamination, and changing lighting conditions [5].

Hyperspectral imaging (HSI) technology captures sequential spectral reflectance at each pixel, enabling simultaneous analysis of the composition and structural characteristics of the target material. This “joint spectral-spatial feature extraction” characteristic makes HSI highly sensitive to micro-cracks on eggshells. In crack regions, stress concentration and microstructural damage alter the local optical properties (reflection, absorption, and transmission), resulting in shifts in reflectance at specific wavelengths or changes in spectral shape. Consequently, even cracks that are nearly invisible under visible light can be detected in the near-infrared or short-wave infrared region [6]. Previous studies have shown that HSI outperforms conventional RGB imaging in agricultural product defect detection, offering significant advantages in both accuracy and robustness [7,8]. For instance, Huang et al. (2021) integrated hyperspectral spectral features with texture features in nectarine quality assessment [9]. Using a Least Squares Support Vector Machine model, they achieved simultaneous identification of external defects and prediction of soluble solids content [9]. Xu et al. (2023) integrated HSI in the range of 866.4–1701 nm with an attention-based convolutional neural network to classify corn seed defects, reaching over 90% accuracy with the optimal model [10]. In the field of non-destructive detection of eggshell cracks, Ahmed et al. systematically reviewed recent advances in optical sensing technologies for the egg industry and emphasized the potential of combining HSI with deep learning to improve crack detection accuracy and enable automated grading [11]. Yao et al. proposed a method that captures images at different wavelengths via HSI to detect cracked eggshells; however, crack tips that are too thin remain difficult to detect due to minimal brightness contrast with surrounding areas [12]. Han et al. employed visible–near-infrared spectroscopy for egg sorting and crack detection, finding that eggshell color differences significantly affect spectral data acquisition, with the high reflectance of white shells causing spectral information loss and reduced detection accuracy [13]. Chen et al. integrated HSI with band selection and deep learning algorithms to develop the HEDIT system, capable of real-time operation on production lines [14]. So et al. utilized visible–near-infrared surface HSI to capture multi-dimensional spectral data from both the eggshell surface and interior. By combining multivariate analysis with pattern recognition algorithms, they realized non-destructive and high-precision microcrack detection, providing crucial technical support for replacing manual inspection and enhancing automated grading [15].

Although HSI combined with machine learning or shallow neural networks can achieve object-level localization of eggshell cracks, such coarse detection remains inadequate for industrial grading applications. Accurate quality control requires not only identifying the presence of cracks but also evaluating their severity, spatial extent, and distinction from surface artifacts (e.g., blood spots and calcification lines). These objectives can only be fulfilled through pixel-level segmentation, which enables precise delineation of crack boundaries and regions, and provides morphological details that serve as quantitative evidence for grading and safety evaluation—information that object-level localization cannot deliver. Despite these advances, several key challenges remain. Variations in shell color and complex backgrounds often lead to feature confusion and missed detections. Shallow models relying on hand-crafted features struggle to integrate joint spectral-spatial feature extraction and to represent the subtle characteristics of microcracks. Furthermore, the high-dimensional and nonlinear nature of hyperspectral data frequently results in information loss in shallow or single-task models, thereby constraining detection sensitivity and overall accuracy.

With the advancement of deep learning, three-dimensional convolutional neural networks (3D-CNNs) have shown advantages in hyperspectral image analysis as they can simultaneously process spatial and spectral information. 3D-CNNs enhance the detection of minute defects while preserving spectral continuity and spatial texture features. Li et al. systematically reviewed 3D-CNN-based hyperspectral image classification approaches, emphasizing their advantage in spectral–spatial feature extraction [16]. Compared with one-dimensional or two-dimensional CNNs, 3D-CNNs significantly reduce information loss and perform excellently in detecting subtle defects such as plant disease identification [17]. Zhang et al. incorporated 3D-CNN and deformable convolution in agricultural product crack detection, further optimizing spectral feature processing to suit the detection of subtle defects such as produce cracks [18]. However, in eggshell crack detection, the standard 3D-CNN still faces limitations: complex background noise, shell color variation, and gradient vanishing or feature degradation as network depth increases, all of which reduce detection accuracy.

To address these issues, this paper proposes a non-destructive eggshell crack detection method that integrates HSI with an improved 3D-CNN to fully exploit joint spectral–spatial features, thereby addressing the limitations of conventional methods in microcrack recognition. Specifically, within the 3D-CNN framework, we incorporate a lightweight SimAM attention mechanism to enhance the response to key spectral–spatial crack regions while suppressing background noise. Additionally, residual modules are adopted to replace standard convolution layers, mitigating gradient attenuation and feature degradation in deeper networks and thus balancing detection accuracy with computational efficiency. Experimental results under varied shell colors and complex background conditions confirm that the proposed method achieves high-precision crack detection and accurate localization, offering a feasible technical pathway for online egg quality grading and intelligent inspection.

## 2. Materials

### 2.1. Sample Preparation

**Figure 1:**
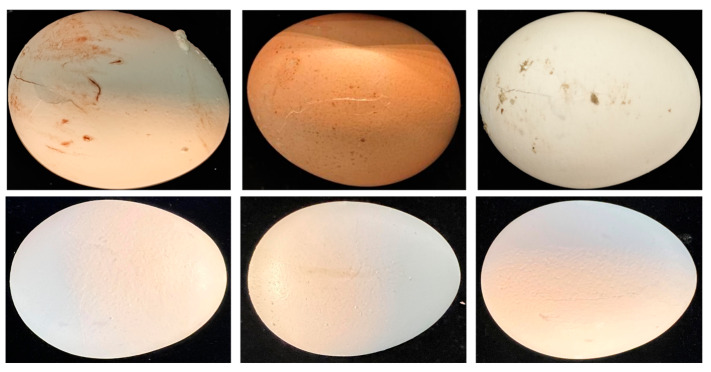
Representative eggs of different varieties and corresponding cracks.

A total of 400 fresh eggs were procured from multiple farmers’ markets and large chain supermarkets in Wuhan, Hubei Province, China, to encompass a diverse range of varieties, shell colors, and production batches, thereby enhancing the dataset’s representativeness. To ensure model robustness, the dataset was constructed to include eggs exhibiting various surface contaminants. After collection, the samples were acclimatized at room temperature (25 ± 2 °C; relative humidity: 60 ± 5%) for 2 h to eliminate the effects of transportation and environmental fluctuations. To simulate realistic damage scenarios, a specialized crack-induction device was designed. Specifically, a customized slider with a mass of 150 g was used on a smooth inclined plane (length: 24 cm). The release height was adjusted between 10 cm and 15 cm to avoid excessive loading while ensuring consistent crack initiation. This produced impact velocities of approximately 1.40–1.71 m/s, corresponding to impact energies of 0.15–0.22 J. To ensure reproducibility across samples, post-impact inspection was performed according to predefined criteria; eggs exhibiting shell crushing or insufficient crack formation were excluded from subsequent analysis. By adjusting the mass and height, various crack types were induced, including visible fractures, star-shaped radial cracks, and fine linear cracks. Representative samples are presented in Figure 1. Specifically, the top row depicts cracks with prominent morphological features that are readily distinguishable, whereas the bottom row illustrates ultra-subtle hairline micro-cracks characterized by minimal contrast and marginal visibility to the unaided eye. This methodology ensured the generation of a diverse and realistic crack dataset, closely mimicking damage incurred during actual transport and handling.

### 2.2. Hyperspectral Data Acquisition

**Figure 2:**
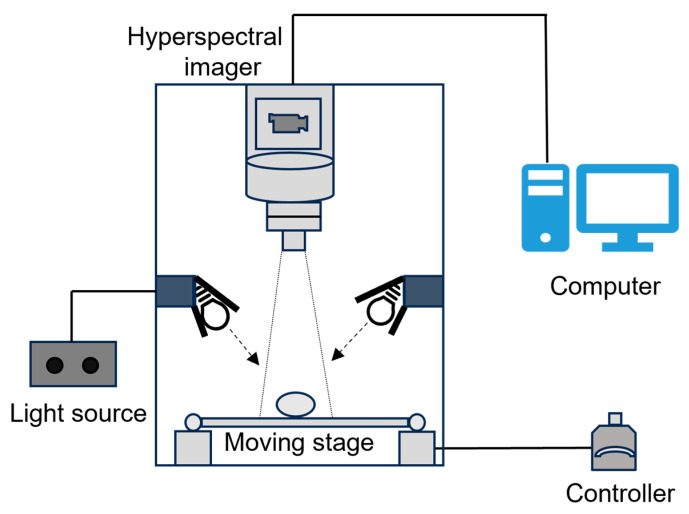
Schematic diagram of the hyperspectral image acquisition system.

As shown in Figure 2, the HSI system used in this experiment consisted primarily of a hyperspectral camera, halogen light sources, a motorized translation stage, and a control computer. The hyperspectral imaging system used in this study was a Specim SWIR camera (Spectral Imaging Ltd., Oulu, Finland) equipped with a Stirling-cooled MCT detector. The system covers a spectral range of 1000–2500 nm with a spectral sampling interval of 5.6 nm and a spectral resolution of 10 nm, yielding 273 spectral bands. The spatial dimension consists of 384 pixels with a pixel size of 24 μm × 24 μm. An F/2.0 fore objective lens was used for image acquisition. During data collection, the exposure time was set to 3.00 ms, and the frame rate was maintained at 50.00 fps to ensure high-quality spectral image reconstruction. The illumination system consisted of two sets of halogen lamps symmetrically positioned at approximately 45° angles on either side of the sample to provide uniform and stable lighting to the imaging area. The egg samples were placed at the center of the motorized stage, which was covered with black velvet to minimize background reflection interference. In this experimental setup, eggs were manually positioned to ensure that the cracked regions were directly exposed to the camera’s field of view. Given that the spectral reflectance of the curved eggshell varies between direct and oblique angles, this orientation was necessary to capture consistent and intrinsic spectral signatures for model training. While this study utilizes a static acquisition protocol, in industrial scenarios, this requirement would be naturally met by roller conveyor systems that rotate the egg to present the entire surface to the sensor. Driven by a stepper motor, the stage moved linearly at a constant speed, while the camera operated in push-broom mode to sequentially capture spatial and spectral information line-by-line, ultimately producing a three-dimensional hyperspectral data cube (Hypercube) that encompassed the entire crack region.

In the current laboratory configuration, a single scanning pass requires approximately 10 s to ensure high spectral resolution. However, it is important to note that the system’s push-broom design enables high spatial flexibility and batch processing: multiple eggs can be arranged in a linear or matrix array within the field of view for simultaneous imaging. Consequently, the scan duration is amortized across all samples, significantly reducing the effective acquisition time per egg. While the current speed is limited by the precision settings of the research-grade translation stage, the imaging sensor supports significantly higher frame rates, indicating that the system is adaptable to continuous high-speed scanning on industrial roller conveyors.

Considering the presence of dark current noise and non-uniform illumination in the hyperspectral system, all raw data were subjected to black–white calibration to obtain relative reflectance [19]. To correct for dark current noise and illumination non-uniformity, standard black–white reference calibration method was applied in this study, and the calculation formula is expressed as follows:

(1) $$I=\frac{I_{0}-D}{W-D}$$

where I denotes the calibrated spectral reflectance; $I_{0}$ represents the reflected intensity of the hyperspectral image; W corresponds to the reflected intensity of the full white calibration image (white reference), and D represents the reflected intensity from the full black calibration image (dark reference).

### 2.3. Egg Crack Annotation

**Figure 3:**
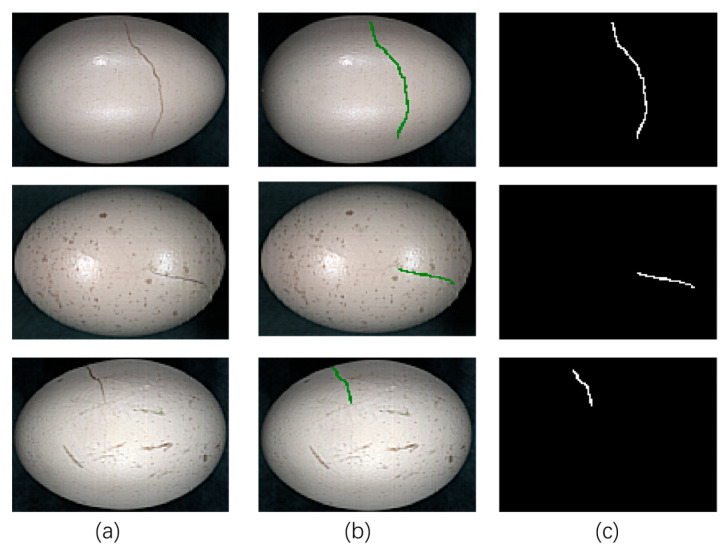
Illustration of the data annotation process: (**a**) original false-color image; (**b**) pixel-wise annotation via the ROI tool, where the green area denotes cracks; (**c**) the resulting binary mask, in which cracks are represented in white.

Given the complex and irregular morphology of eggshell cracks, pixel-level manual annotation was performed to ensure accurate ground-truth labels (Figure 3). The annotation process was carried out using ENVI 5.3 software (ITT Visual Information Solutions, Inc., in Boulder, CO, USA), which features a Region of Interest (ROI) tool that supports pixel-level operations. Specifically, a false-color composite image of each hyperspectral cube was first generated. Using the Region of Interest (ROI) tool, annotators manually traced crack boundaries pixel by pixel. Crack regions were assigned a value of 1, and non-crack regions (including intact shell and background) were assigned 0, producing a binary mask for each sample. Each calibrated hypercube was thus paired with a corresponding mask, forming the image-label pairs for subsequent model training and evaluation.

## 3. Methodology

### 3.1. Selection of Hyperspectral Feature Bands

Hyperspectral data are characterized by high dimensionality, strong inter-band correlation, and significant information redundancy. Effective band selection is crucial to mitigate the “curse of dimensionality,” reduce computational cost, and improve model generalization. This study employed three complementary band selection methods which apply distinct constraints from the perspectives of variable importance, collinearity minimization, and global optimization, respectively. This multi-strategy approach ensures the selected feature bands are both informative and non-redundant. Three band selection algorithms—CARS, SPA, and RF—were evaluated. Based on a preliminary assessment of their performance, the CARS algorithm was chosen for final model development due to its superior effectiveness in identifying the most informative spectral bands. A detailed quantitative comparison of the three methods is presented in Section 4.2 to further justify the selection.

(1) Competitive Adaptive Reweighted Sampling (CARS)

CARS simulates the “survival of the fittest” process by iteratively eliminating redundant variables during the selection procedure. The core principle involves using Partial Least Squares Regression (PLSR) to evaluate the importance of each spectral band, while employing an exponential decay mechanism to control the number of retained variables [20]. The weighting coefficient for each band is calculated as:

(2) $$w_{j}=\frac{\left|\beta_{j}\right|}{\sum_{i=1}^{p}|\beta_{i}|}$$

where $\beta_{j}$ represents the regression coefficient of the j-th band in the current PLSR model, and p denotes the total number of bands. These weights are used for weighted sampling, retaining the most important variables. Finally, CARS retains the subset of bands that minimizes the cross-validation error, thereby enhancing prediction accuracy and robustness.

(2) Successive Projections Algorithm (SPA)

SPA is a forward-selection algorithm designed to minimize multicollinearity among variables by leveraging vector projection operations. It starts by selecting the spectral variable (band) with the largest norm. In each subsequent step, the algorithm projects all remaining bands onto the orthogonal complement of the subspace spanned by the already-selected bands [21]. The band with the maximum projection value (i.e., the largest amount of novel information) is then added to the feature set. The projection operation is defined as:

(3) $$Px_{j}=x_{j}-X_{s}(X_{s}^{T}X_{s}{)}^{-1}X_{s}^{T}x_{j}$$

where $x_{j}$ is the vector of the j-th remaining band, $X_{s}$ is the matrix of already-selected bands, and $Px_{j}$ is the projection residual. SPA iterates until a predefined number of bands are selected, effectively constructing a set of variables with minimal redundancy.

(3) Random Frog (RF)

The RF algorithm is a global search method inspired by reversible-jump Markov Chain Monte Carlo (MCMC), which effectively explores the feature space to avoid local optima [22]. It works by iteratively generating and evaluating candidate band subsets. The basic procedure involves generating a new subset S′ through random operations such as replacing, adding, or removing spectral bands. The subset is accepted based on a performance metric. The acceptance probability is given by:

(4) $$P\left(accept\right)=\min\left(1,\exp\left(-\frac{E\left(S^{\prime }\right)-E\left(S\right)}{T}\right)\right)$$

where E(S) and E(S′) represent the cross-validation errors of models built on the current subset and the candidate subset, respectively. T is the temperature parameter that decreases over iterations, controlling the exploration-exploitation trade-off. After numerous iterations, the frequency of each band appearing in high-performing subsets is recorded and used as the final selection criterion. This approach can identify bands with limited individual contribution but significant synergistic effect when combined, thereby enhancing the model’s ability to capture complex spectral characteristics.

### 3.2. Proposed 3D-CrackNet Model

Conventional 3D-CNNs can simultaneously extract spatial–spectral features from hyperspectral images (HSI) but face two primary limitations: (1) gradient vanishing and network degradation with increasing depth, which hinders the learning of complex features [23]; and (2) insufficient feature discrimination, as standard convolutions treat all features equally, making it difficult to distinguish subtle cracks from complex backgrounds [24]. To overcome these challenges, we propose 3D-CrackNet, a deep learning framework that integrates residual learning and a parameter-free attention mechanism to enhance feature representation and segmentation accuracy for eggshell cracks.

#### 3.2.1. Overall Architecture

**Figure 4:**
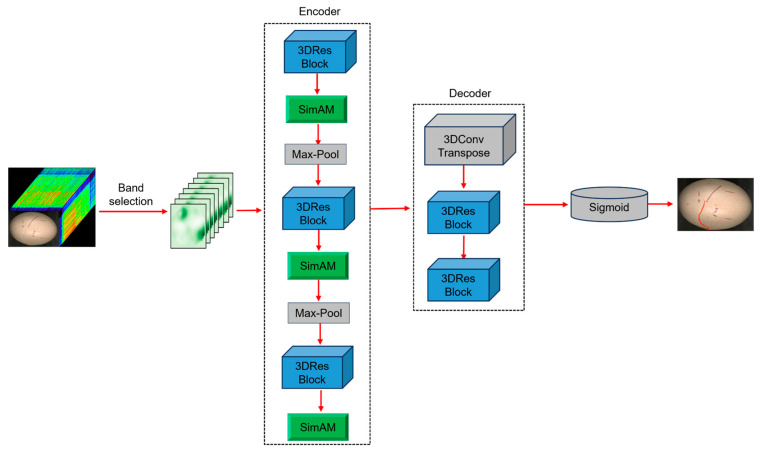
Overall architecture of the proposed 3D-CrackNet model.

As illustrated in Figure 4, 3D-CrackNet adopts a U-Net-like encoder–decoder architecture tailored for pixel-wise hyperspectral image segmentation. The encoder comprises three sequential feature extraction units, each containing a 3D residual block (3D-ResBlock), a SimAM attention module, and a 3D max-pooling layer. This design enables progressive abstraction of the input hyperspectral cube, transforming high-resolution shallow features into low-resolution deep semantic representations. The decoder gradually restores spatial details through upsampling and skip connections, effectively recovering crack boundaries. The network culminates in a sigmoid activation function that outputs a probability map with the same spatial dimensions as the input. This map is subsequently thresholded to produce the final binary crack segmentation, enabling end-to-end training and optimization.

#### 3.2.2. Three-Dimensional Residual Block

**Figure 5:**
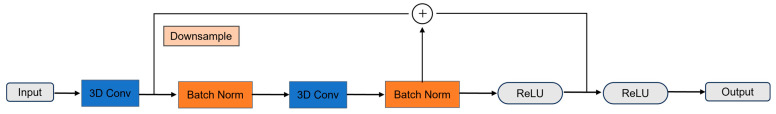
Structure of the 3D residual block (3D-ResBlock).

In hyperspectral egg crack detection tasks, fine cracks often appear as weak and spatially–spectrally discrete anomaly signals that are prone to being diminished or lost during deep network training. To address this issue, the proposed 3D-CrackNet incorporates a three-dimensional convolutional residual block (3D Residual Block), as shown in Figure 5, to jointly model local features in both the spatial and spectral dimensions while employing a residual mechanism to preserve crack-specific features.

The block consists of two sequential 3 × 3 × 3 3D convolutional layers, each followed by Batch Normalization (BN) and a ReLU activation, which enhance the network’s nonlinear representational ability and stabilize the training process. Since egg surface cracks manifest as subtle spectral anomalies and localized texture perturbations in hyperspectral imagery, 3D convolution aggregates contextual neighborhood information in the spatial domain while capturing spectral reflectance variations across different bands in the spectral domain. To mitigate gradient vanishing and information loss in deeper networks, a residual connection is incorporated. Here, the input feature x is added directly to the output of the convolutional main branch via an identity mapping or a downsampling pathway F(x). The final output is formulated as:

(5) $$y=\sigma \left(BN({Conv}_{3D}(BN({Conv}_{3D}(x))))\right)+F(x)$$

where σ denotes the ReLU activation function, which is applied to the output of the preceding batch normalization layer. This structure not only ensures stable training in deep networks but also effectively enhances the fidelity of fine cracks within multi-scale features, enabling more accurate discrimination between intact eggshell regions and those containing cracks. In summary, the 3D Residual Block, by combining spatial–spectral joint modeling with residual enhancement, improves the robust extraction of crack features and provides more discriminative high-level semantic features for subsequent modeling.

#### 3.2.3. SimAM Attention Module

**Figure 6:**
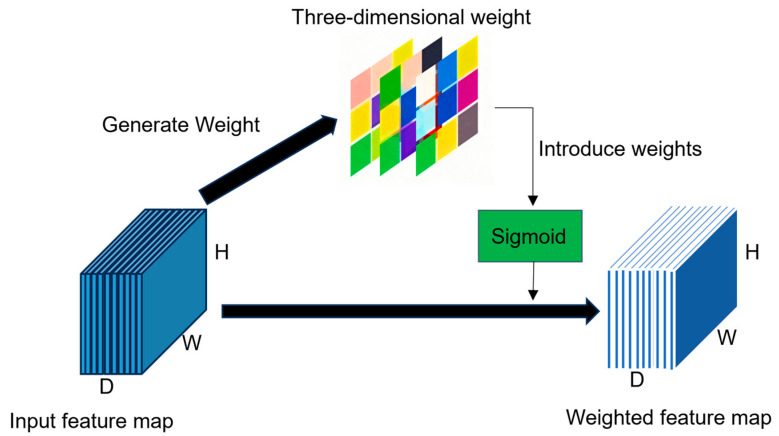
SimAM attention structure.

Within the 3D-CrackNet framework, 3D Residual Block operates synergistically with the SimAM attention module. The 3D-ResBlock extracts joint spatial–spectral features of eggshell cracks through multi-scale convolution while maintaining training stability. The SimAM module then refines these features via fine-grained, voxel-level weighting, which amplifies crack-related responses and suppresses background interference. This complementary mechanism significantly enhances the precision and robustness of hyperspectral crack segmentation. Unlike conventional one-dimensional spectral attention or two-dimensional spatial attention, SimAM assigns a unique three-dimensional weight to each voxel in the input feature map. This capability enables the simultaneous modeling of discriminative information across both spatial dimensions (H, W) and the spectral dimension (D), allowing the model to highlight abnormal spectral reflectance in crack regions while reinforcing spatial continuity in their vicinity. A distinctive advantage of SimAM is its parameter-free design: attention weights are derived directly from a closed-form solution to an energy function, without introducing additional learnable parameters. This results in a lightweight structure with high computational efficiency and ease of integration into various network architectures. The design is inspired by visual neuroscience, where salient features (e.g., abnormal spectral signatures or surface textures) naturally attract attention in biological visual systems, while redundant background information is suppressed. Given an input feature map of size H × W × D, the SimAM module constructs an energy function for each voxel to evaluate its separability from other voxels within the same channel, defined as:

(6) $$E(x_{i})=(y_{g}-g^{*}{)}^{2}+\frac{1}{N-1}\sum_{i=1}^{N-1}(y_{o}-x_{i}^{*}{)}^{2}$$

where $y_{g}$ denotes the response of the target voxel, $g^{*}$ and $x_{i}^{*}$ represent the channel mean and other voxel values within the same channel, respectively, and N is the total number of voxels per channel. This energy function determines the saliency of a voxel by quantifying the difference between the target feature and the surrounding background features. Subsequently, the energy values are normalized using a Sigmoid function to produce a voxel-wise weight matrix, which is applied element-wise to the input feature map to yield the attention-weighted output:

(7) $$X^{\prime }=\sigma (E(X))⊙X$$

where ⊙ denotes element-wise multiplication, and σ represents the Sigmoid function. Compared with one-dimensional or two-dimensional attention mechanisms, SimAM (Figure 6) offers several advantages: (1) Three-dimensional weight assignment, enabling the model to simultaneously account for joint spectral–spatial discriminative characteristics; (2) No additional learnable parameters—analytical (closed-form) computation ensures a lightweight and efficient design; (3) Simple architecture, making it straightforward to integrate into existing networks. Leveraging this module, 3D-CrackNet can effectively highlight the abnormal spatial–spectral signatures of egg crack regions, thereby improving detection sensitivity and discriminative capability.

### 3.3. Experimental Setup and Evaluation Metrics

The experiments were implemented in PyTorch 2.1.0 with CUDA 11.8 acceleration on a workstation featuring an NVIDIA GeForce RTX 3090 GPU and an Intel Core i7-13700KF CPU. The training procedure spanned 100 epochs with a batch size of 32, utilizing the AdamW optimizer at an initial learning rate of 0.0001. A Cosine Annealing Warm Restarts scheduler was used to adjust the learning rate dynamically, thereby balancing training efficiency and final performance. To mitigate the class imbalance issue in crack segmentation, the Focal Dice Loss was chosen as the criterion for model optimization.

A total of 400 hyperspectral egg samples were collected and randomly divided into training (320 samples), validation (40 samples), and testing (40 samples) subsets at a ratio of 8:1:1. To mitigate the risk of overfitting, regularization was integrated through the training process and model architecture. An L2 weight decay term (λ = 1 × 10^−4^) was applied within the AdamW optimizer to penalize large weights. Furthermore, the inclusion of batch normalization layers in the 3D-CNN architecture helped stabilize training and act as an implicit regularizer. Most importantly, an early stopping strategy was adopted, which halted training if the validation loss did not improve for 15 consecutive epochs, thereby preventing the model from over-optimizing on the training data. These strategies collectively enhanced the model’s ability to generalize to unseen samples.

Since crack pixels occupy only a small proportion of the overall image, conventional overall accuracy metrics cannot adequately evaluate crack recognition performance. Therefore, a multi-dimensional evaluation strategy was adopted in this study. Based on the confusion matrix, four standard metrics—Intersection over Union (IoU), Precision, Recall, and F1-Score—were computed to comprehensively assess segmentation performance. In addition, the model’s training time was recorded to evaluate computational efficiency in practical applications.

(8) $$IOU=\frac{TP}{TP+FP+FN}$$

(9) $$Precision=\frac{TP}{TP+FP}$$

(10) $$Recall=\frac{TP}{TP+FN}$$

(11) $$F1\text{-score}=\frac{2TP}{2TP+FP+FN}$$

Here, TP denotes the number of positive samples correctly predicted by the model; FP denotes the number of negative samples incorrectly predicted as positive; and FN denotes the number of positive samples incorrectly predicted as negative.

## 4. Experimental Results and Analysis

### 4.1. Spectral Feature Analysis of Intact and Cracked Targets

**Figure 7:**
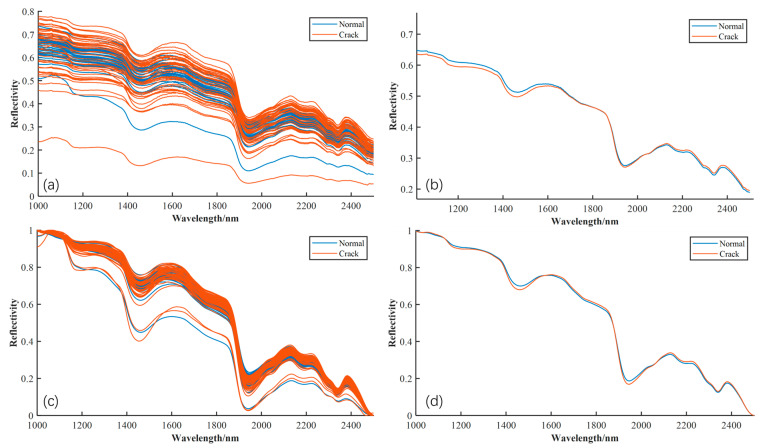
Spectral profiles of intact and cracked eggshells: (**a**) original spectra; (**b**) mean original spectra; (**c**) spectra after SG + MSC preprocessing; (**d**) mean spectra after SG + MSC preprocessing.

A systematic analysis of hyperspectral reflectance data from cracked and intact eggshell regions was conducted. Figure 7a illustrates the raw spectral curves for the two sample types in the 1000–2500 nm wavelength range. As observed, the raw data exhibit baseline drift and scattering effects, leading to considerable intra-class variation. To highlight the overall trend, the mean spectra for each class were calculated, as shown in Figure 7b. The spectral shapes demonstrate high consistency with significant overlap, with only slight variations in reflectance intensity. Specifically, cracked eggshells generally exhibit lower reflectance than intact eggshells, yet the overall spectral pattern remains consistent across all bands. To enhance spectral quality and accentuate subtle differences, the raw spectral data were pre-processed using the Savitzky–Golay (SG) smoothing algorithm, combined with Multiplicative Scatter Correction (MSC) [25,26]. The results of this preprocessing are shown in Figure 7c, where the spectral curves are more compact and concentrated. This indicates that the preprocessing method effectively reduces baseline drift, scattering effects, and other non-chemical interferences. As a result, the mean spectral curve in Figure 7d is smoother and more standardized.

From a biochemical perspective, spectral characteristics at different wavelengths are closely tied to the primary chemical constituents of the eggshell and its underlying structures. Considering that the average eggshell thickness is approximately 0.30–0.40 mm, the incident near-infrared light (1000–2500 nm) exhibits sufficient penetration depth to pass through the calcified layer and interact with the shell membrane and outer albumen [27,28]. Consequently, the recorded spectra represent a composite signal of the shell and subsurface components. Previous studies have demonstrated that the absorption valleys near 1450 nm and 1940 nm correspond to the first and second overtone water absorption bands [29,30], which are detectable due to the presence of bound moisture in the underlying organic membrane and albumen. Although the calcified shell itself contains limited water, micro-cracks may increase the optical contribution of the exposed membrane, thereby influencing reflectance in these bands. Furthermore, the region between 2100 and 2300 nm has been linked to combination absorptions of C=O and N–H functional groups associated with the protein-rich eggshell membrane [31]. Micro-cracks can alter the scattering path and partially expose this membrane material, resulting in subtle spectral differences within this range. Additionally, near-infrared scattering features related to the crystalline CaCO_3_ structure may also be affected by microstructural disruption of the shell.

Despite the preprocessing, the spectral curves of both classes still exhibit significant overlap across most wavelengths, indicating weak separability. Despite the preprocessing, the spectral curves of both classes still exhibit significant overlap across most wavelengths, indicating weak separability. The main challenge in hyperspectral crack detection lies in the high spectral redundancy across the broad 1000–2500 nm wavelength range, where adjacent bands are strongly correlated, with many contributing minimally to biochemical discrimination. This redundancy in-creases the computational complexity of data processing and model training and can obscure subtle spectral variations caused by cracks.

### 4.2. Feature Band Extraction and Performance Analysis for Crack Detection

Given the spectral redundancy and weak separability identified in Section 4.1, three feature band selection methods—CARS, SPA, and RF—were applied to reduce dimensionality, mitigate multicollinearity, and enhance crack detection performance.

(1) Feature Band Selection Using the CARS Algorithm

**Figure 8:**
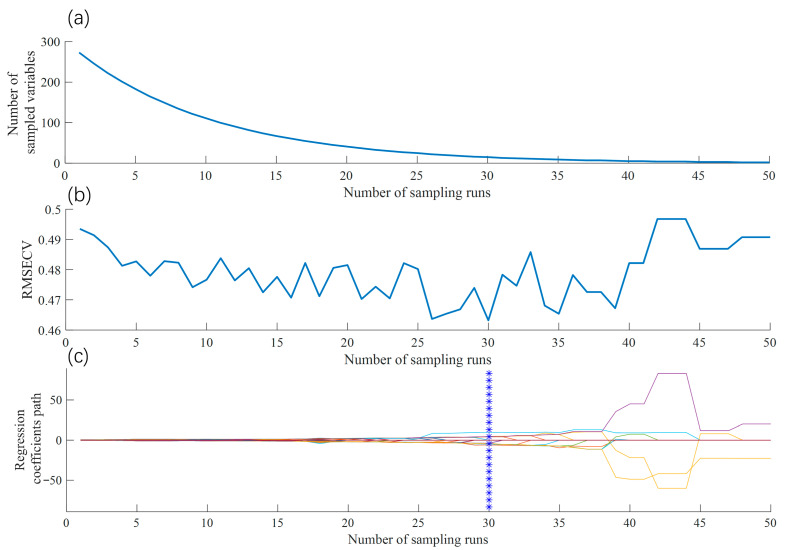
Feature wavelengths selection using the CARS method: (**a**) number of selected feature wavelengths; (**b**) RMSECV values across iterations; (**c**) regression coefficient paths, where each colored curve represents the change in the regression coefficient of a specific wavelength variable across sampling iterations; the blue vertical dashed line indicates the optimal number of sampling runs selected based on the minimal RMSECV.

For the CARS algorithm, the number of iterations was set to 50, and the sampling ratio was set to 0.8. As illustrated in Figure 8, the feature selection process significantly reduces the number of wavelengths. In Figure 8a, the initial count of approximately 280 wavelengths rapidly decreases as iterations progress, indicating the efficient removal of redundant and irrelevant features. Figure 8b shows that the RMSECV, calculated via Monte Carlo cross-validation (MCCV), decreases steadily until reaching a minimum at the 30th iteration, suggesting optimal predictive performance at this point. After the 30th iteration, the RMSECV slightly increases as some critical features are inadvertently discarded. The regression coefficients in Figure 8c confirm that the 30th iteration marks the optimal selection, resulting in the identification of 15 key wavelengths.

(2) Feature Band Selection Using the SPA Algorithm

**Figure 9:**
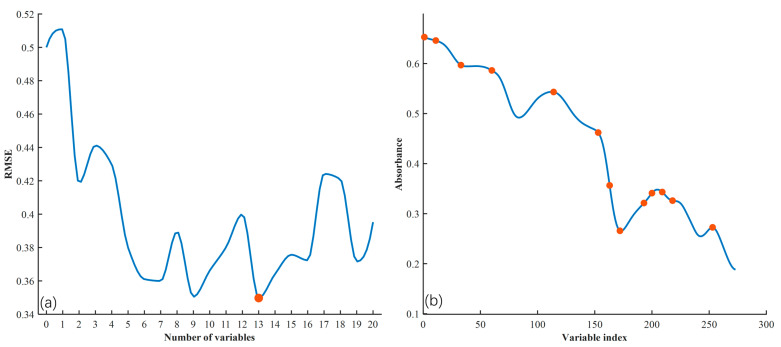
Feature wavelength selection using the SPA method: (**a**) RMSE versus number of wavelengths; (**b**) distribution of selected wavelengths.

For the SPA algorithm, the maximum number of wavelengths was set to 20. As shown in Figure 9a, the RMSE value decreases sharply at first as more wavelengths are added, then plateaus, indicating the elimination of redundant information. For the SG + MSC preprocessed spectral data, 13 key wavelengths were selected, achieving the lowest RMSE and optimal predictive accuracy, as shown in Figure 9b. These 13 wavelengths cover multiple crucial regions of the spectrum, providing a comprehensive representation of the samples’ spectral characteristics.

(3) Feature Band Selection Using the RF Algorithm

**Figure 10:**
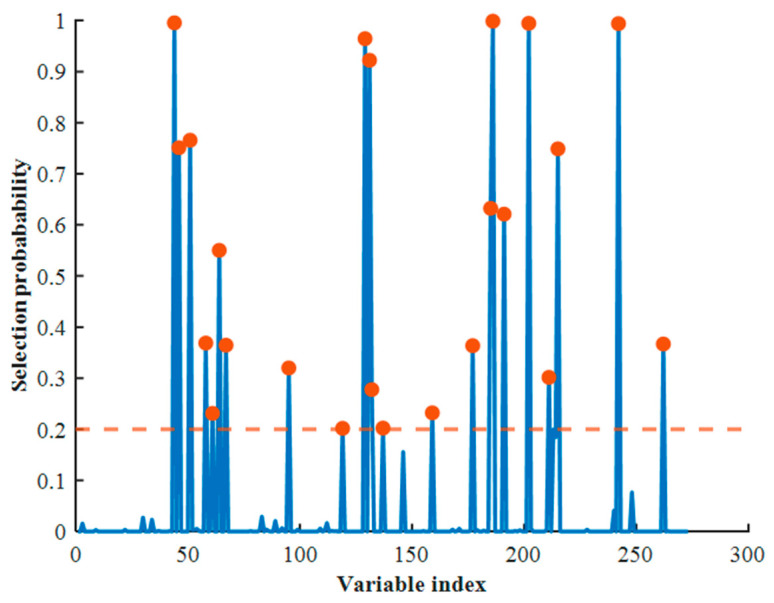
Feature wavelength selection using the RF method: probability distribution of selected wavelengths.

The RF algorithm obtains the selection probability distribution of each wavelength variable through multiple iterations of sampling (Figure 10). The horizontal axis represents the full-spectrum wavelength index, while the vertical axis denotes the probability of a variable being selected. To identify highly important bands, a selection probability threshold of 0.2 was set, with variables exceeding this threshold considered to be strongly correlated with the modeling objective. The RF method ultimately selected 23 key wavelengths, providing an alternative feature selection approach.

(4) Method Comparison and Performance Evaluation

To evaluate the effectiveness of the three feature selection methods, the selected wavelengths from CARS, SPA, and RF were used as model inputs and compared with the full-band input. The results, summarized in Table 1, demonstrate that the CARS method outperforms all other methods across all evaluation metrics. Specifically, the IoU and F1 scores for CARS reached 59.99% and 74.99%, respectively—an improvement of approximately 8.2% and 6.8% over the full-band input—while reducing training time to 35.5 min. Despite a reduction of over 90% in data dimensionality, CARS maintained or even improved segmentation performance, effectively eliminating noise and redundancy while preserving the most discriminative features. In contrast, while the SPA method offers a training efficiency advantage, its accuracy metrics are slightly lower than those of CARS. The RF method performed well in terms of Precision but had a low Recall, resulting in overall performance inferior to both CARS and SPA. The full-band input method achieved the lowest scores across all metrics and incurred significantly higher training costs. In conclusion, similar to the spectral feature analysis in Section 4.1, the results from this feature band selection process demonstrate that the CARS method strikes the optimal balance between accuracy and efficiency. Therefore, the feature bands selected by CARS were adopted as model inputs for all subsequent experiments.

### 4.3. Ablation Experiment

To evaluate the individual contributions of the core components—3D-ResBlock and the SimAM attention mechanism—in the proposed 3D-CrackNet model, a comprehensive ablation study was conducted. A standard 3D-CNN served as the baseline. The performance impact of each component was assessed by progressively integrating them into the baseline model. All models were trained and tested under identical conditions using the 15 feature bands selected by the CARS method. To statistically validate the performance improvements, a paired t-test was performed on the evaluation metrics (IoU, Precision, Recall, F1-score) obtained from five independent runs of each model configuration. The results are summarized in Table 2.

The baseline 3D-CNN model achieved IoU, Precision, Recall, and F1-score values of 52.08%, 71.09%, 66.07%, and 68.49%, respectively, serving as a reference for subsequent performance improvements. With the integration of the SimAM attention mechanism, the IoU increased to 53.95%, and Precision rose to 72.70%. Statistical analysis confirmed that the improvement in Precision was statistically significant (*p* < 0.05), indicating that this mechanism effectively enhances feature representation and spatial dependency modeling, thereby improving feature extraction performance. When the ResBlock module was incorporated, the IoU improved to 56.77%, and the F1-score increased to 72.42%. Both of these improvements were found to be statistically significant (*p* < 0.05), demonstrating its positive effect in strengthening deep feature propagation and mitigating gradient vanishing. By integrating both SimAM and ResBlock to form the complete 3D-CrackNet model, overall performance reached its peak: IoU, Precision, Recall, and F1-score achieved 60.62%, 75.09%, 75.89%, and 75.49%, respectively. These represent improvements of 8.54%, 4%, 9.82%, and 7% over the baseline model. All of these improvements over the baseline model were statistically significant (*p* < 0.05). Despite the increase in model parameters and training time, the performance gains significantly outweighed the additional computational cost, reflecting the model’s rational and efficient design. In conclusion, the ablation results fully demonstrate the complementarity and critical roles of ResBlock and SimAM. The complete 3D-CrackNet model significantly outperforms other combinations of components in terms of accuracy, recall, and overall performance, achieving an excellent balance between performance improvement and computational complexity. This validates the effectiveness and practical value of the proposed model for hyperspectral egg crack segmentation tasks.

### 4.4. Comparative Experiment

To further validate the structural advantages of 3D-CrackNet in spatial–spectral feature extraction and crack detection, a series of comparative experiments were conducted with several representative convolutional neural network models as benchmarks. All models used the 15 feature bands selected by the CARS method as input, and training environments, hyperparameter settings, and data preprocessing were kept consistent. The 1D-CNN model classified solely based on pixel-level spectral vectors, ignoring spatial contextual features. The 2D-CNN model took single-band images as input and relied on spatial information, but it almost entirely lost spectral dimension features. The 3D-CNN (baseline) model jointly leveraged spatial and spectral information to achieve integrated spatial–spectral modeling. Building upon the 3D-CNN, the proposed 3D-CrackNet model incorporates a ResBlock and the SimAM attention mechanism to further enhance spatial–spectral feature representation.

**Figure 11:**
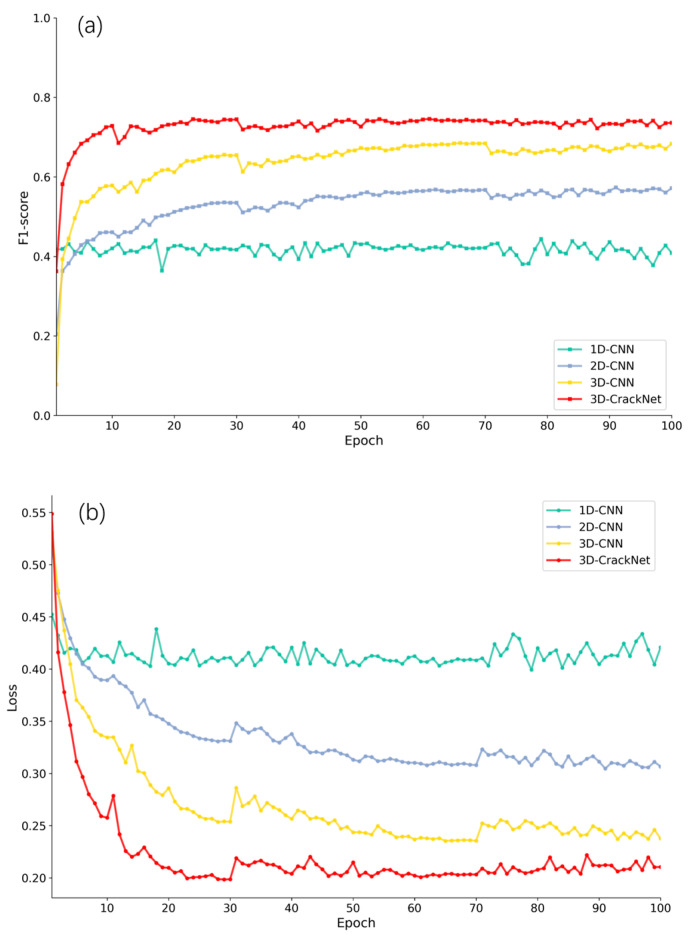
Training convergence curves and loss decline curves of different CNN models: (**a**) Trend of F1-score with Epochs; (**b**) Trend of loss with Epochs.

As shown in the training curves (Figure 11), 3D-CrackNet converges to a stable and significantly higher F1-score (approximately 0.75) after about 25 epochs, while also exhibiting the most rapid loss reduction and the lowest final loss value. According to the quantitative results in Table 3, the IoU of the 1D-CNN is only 29.29%, the lowest among all models, suggesting that relying solely on spectral information is insufficient for crack detection. With the incorporation of spatial information, the 2D-CNN shows a marked improvement over the 1D-CNN, achieving an IoU of 40.37% and an F1-score of 57.33%. However, due to the absence of spectral dimension features, it still suffers from significant limitations. The 3D-CNN, which simultaneously leverages spatial and spectral information, significantly outperforms the previous two models, attaining an IoU of 52.51% and an F1-score of 68.86%. Building upon this, 3D-CrackNet achieves a further breakthrough, with IoU and F1-score increasing to 60.62% and 75.49%, respectively—representing improvements of 8.11% and 6.63% over the baseline 3D-CNN. These results place 3D-CrackNet’s performance among the leading results in deep learning-based crack detection.

The fundamental reason for the performance enhancement lies in the ResBlock’s ability to mitigate network degradation and strengthen deep feature propagation, while the SimAM attention mechanism allows the model to focus on small crack regions, thereby significantly improving its capability to detect subtle defects. Based on the comparative results and training curve analysis, it is evident that 3D-CrackNet demonstrates significant advantages in joint spatial–spectral feature representation and crack segmentation tasks using hyperspectral data, surpassing common CNN models in both accuracy and stability. This fully validates its effectiveness and feasibility for practical applications.

### 4.5. Visualization Analysis of Crack Detection Results

**Figure 12:**
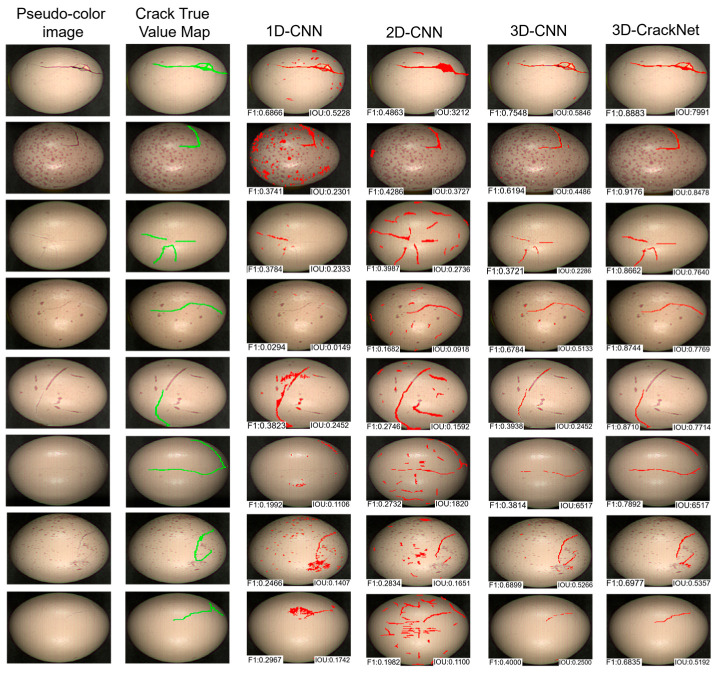
Visual comparison of crack segmentation results (Green: Ground Truth; Red: Model Predictions). From left to right: Original image, Ground Truth (GT), 1D-CNN, 2D-CNN, 3D-CNN, and 3D-CrackNet.

To visually evaluate the performance of the enhanced 3D-CrackNet model in egg crack detection tasks, representative samples were selected for comparative visualization analysis. The comparison involved three models: the 1D-CNN, which relies solely on spectral information, and the 2D-CNN, which utilizes only spatial information. The results are shown in Figure 12, where green lines represent the true crack positions (Ground Truth), and red regions denote the predicted outputs of each model. To provide a quantitative reference for these visual comparisons, the instance-level F1-score and Intersection over Union (IoU) metrics for each prediction are annotated at the bottom of the corresponding sub-images.

As observed in the figure, the 1D-CNN, which depends exclusively on spectral information, struggles to capture spatial structural features. It tends to produce incomplete detections and false alarms, particularly for slender and low-contrast cracks, often misclassifying background spots as cracks.(e.g., resulting in a low IoU of 0.2301 for the spotted egg in the second row) The 2D-CNN, by leveraging spatial context, depicts crack shapes more accurately; however, due to its neglect of fine-grained spectral features, it fails to detect smaller cracks and generates false positives in regions with complex textures. The 3D-CNN model, which integrates both spectral and spatial information, shows clear improvements over both the 1D-CNN and 2D-CNN, yet some missed and false detections still remain. In contrast, the 3D-CrackNet model effectively integrates multi-dimensional information through joint spatial–spectral modeling, which is crucial for distinguishing structural cracks from superficial interferences. The ResBlock module ensures stable deep feature propagation, while the SimAM attention mechanism dynamically weights discriminative spectral–spatial characteristics, thereby specifically enhancing crack-related features and suppressing responses from irrelevant background variations such as stains and natural shell textures. Quantitative analysis of the representative samples displayed in Figure 12 further confirms these visual observations. Specifically, for challenging scenarios with complex spotted backgrounds (Row 2), while the comparative models struggled with IoUs below 0.45, 3D-CrackNet achieved a remarkable IoU of 0.8478 and an F1-score of 0.9176. Across all displayed samples, the proposed model consistently maintained high metric scores (e.g., F1 > 0.68 and IoU > 0.51), significantly outperforming the baseline models. As a result, the predictions exhibit optimal performance in terms of crack contour integrity, positional accuracy, and robustness against false positives caused by non-crack regions. The model successfully segments fine cracks while effectively avoiding the misclassification of eggshell spots, stains, or texture patterns as cracks, demonstrating a clear advantage in handling real-world variability.

In summary, the visualization results in Figure 12 clearly highlight the superiority of pixel-level segmentation. They not only validate the robustness and accuracy of 3D-CrackNet under complex background conditions but also underscore why precise pixel-wise delineation is essential for reliable and quantitative assessment of eggshell cracks—something that object-level detection alone cannot achieve. The visualization results corroborate the quantitative findings, demonstrating that 3D-CrackNet provides a robust and precise solution for automated, pixel-level egg crack inspection, which is essential for reliable quality assessment.

## 5. Discussion

This study addresses the application requirements of HSI in non-destructive egg crack detection by proposing the 3D-CrackNet model, which integrates feature band selection, deep spatial–spectral fusion, and a lightweight attention mechanism for high-precision recognition of fine cracks. Comprehensive experimental results demonstrate that the proposed method substantially improves detection accuracy, robustness, and generalization capability compared to existing models. These findings not only validate the effectiveness of individual techniques but also highlight several key scientific challenges and technical principles involved in integrating hyperspectral data with deep learning models for agricultural and food inspection applications.

At the dataset level, hyperspectral imagery, despite its rich spectral information, faces challenges such as high-dimensional redundancy, noise sensitivity, and the “curse of dimensionality.” These issues make the rational selection of feature bands critical to model performance. In this study, dimensionality reduction was achieved using the CARS algorithm, which retained spectral channels strongly correlated with crack defects, thus improving both computational efficiency and recognition accuracy. This indicates that data-driven feature compression is not only an effective means for algorithm acceleration but also crucial for enhancing a model’s generalization ability. In future efforts to construct larger-scale, multi-variety, and multi-condition datasets, band selection and feature refinement will remain central steps for improving model stability and performance.

At the methodological level, 3D-CrackNet demonstrates the efficacy of joint spatial–spectral modeling for crack detection. Traditional classification methods such as Botta et al. [5] achieve high accuracy by making a single binary decision per egg. In contrast, 3D-CrackNet performs pixel-wise segmentation—a more challenging task that requires precise localization of crack boundaries. Although the pixel-wise F1-score (75.49%) is numerically lower than classification accuracy, it provides critical morphological information (e.g., crack dimensions and location) essential for industrial grading. This capability allows differentiation between cosmetic and structural cracks, which binary classification cannot achieve. Thus, while direct metric comparison may be misleading, the proposed method offers superior utility for fine-grained quality control in practical applications.

At the application level, the proposed method provides technical support for automated, non-destructive egg crack detection. Compared with traditional manual inspection or single optical methods, the hyperspectral imaging–deep learning combined approach significantly enhances detection consistency and objectivity. While the current model performs excellently in laboratory settings, further optimization is required in terms of computational efficiency, hardware compatibility, and real-time processing capabilities to meet the demands of large-scale industrial production. Furthermore, to achieve industrial-grade full-shell inspection, the proposed method can be extended to a multi-view system, for instance, by employing a rotary mechanism or a multi-camera array for data acquisition. The core algorithm of this study, 3D-CrackNet, is well-suited for processing multi-view data. The primary subsequent challenge will focus on the fusion of multi-view segmentation results and the generation of a comprehensive defect map.

Finally, while hyperspectral imaging provides rich spectral information, its deployment in industrial production lines may be constrained by hardware cost, system integration complexity, and the need for high-speed acquisition modules. Furthermore, industrial grading requires continuous inspection on conveyor belts, necessitating a total processing time per egg that is significantly faster than achieved here. Although the core 3D-CrackNet model inference shows promise with a latency of approximately 0.9 s, future work must focus on developing a synchronized, high-throughput pipeline. This entails integrating high-speed cameras, real-time preprocessing hardware (e.g., FPGAs), and further model optimization to bridge the gap between laboratory proof-of-concept and commercial-scale application. These factors should be considered when translating the proposed approach into large-scale commercial applications.

## 6. Conclusions

This study presents a non-destructive egg crack detection method based on HSI and the improved 3D-CrackNet model, which integrates the CARS algorithm for feature band selection, a 3D residual module, and the SimAM attention mechanism. The CARS algorithm effectively reduced dimensionality, retaining 15 key bands that enhanced detection accuracy and computational efficiency. Experimental results demonstrate that 3D-CrackNet outperforms 1D-CNN, 2D-CNN, and conventional 3D-CNN models, achieving an F1-score of 75.49% and an IoU of 60.62%. Ablation studies confirmed that the integration of ResBlock and SimAM significantly improved model performance, particularly in fine crack detection under complex backgrounds.

These findings highlight the potential of HSI and deep learning for automated, high-precision crack detection in agricultural products. Future work will focus on further model optimization for real-time industrial application and expanding the detection capabilities to multiple defect categories.

## Figures and Tables

**Table 1:** Performance comparison of different feature band selection methods and full-band input.

	Number of Bands	IOU (%)	Precision (%)	Recall (%)	F1-Score (%)	Training Time (min)
SPA	13	56.74	76.48	68.73	72.4	33.80
CARS	15	60.62	75.09	75.89	75.49	35.52
RF	23	55.05	76.91	65.95	71.01	38.33
Full band	273	51.71	72.66	64.20	68.17	340.10

**Table 2:** Ablation experiment results and performance comparison.

Model	IoU (%)	Precision (%)	Recall (%)	F1-Score (%)	Training Time (min)
3D-CNN	52.08	71.09	66.07	68.49	18.92
3D-CNN + SimAM	53.95	72.70	67.66	70.09	29.35
3D-CNN + ResBlock	56.77	75.61	69.49	72.42	31.38
3D-CrackNet	60.62	75.09	75.89	75.49	35.52

**Table 3:** Performance comparison and analysis of different CNN models.

Model	IOU (%)	Precision (%)	Recall (%)	F1-Score (%)	Training Time (min)
1D-CNN	29.29	53.14	38.25	44.37	8.92
2D-CNN	40.37	57.41	57.26	57.33	10.78
3D-CNN	52.08	71.09	66.07	68.49	18.92
3D-CrackNet	60.62	75.09	75.89	75.49	35.52

## Data Availability

The raw data supporting the conclusions of this article will be made available by the authors on request.
